# Differential Effects of *Viscum album* Preparations on the Maturation and Activation of Human Dendritic Cells and CD4^+^ T Cell Responses

**DOI:** 10.3390/molecules21070912

**Published:** 2016-07-14

**Authors:** Chaitrali Saha, Mrinmoy Das, Emmanuel Stephen-Victor, Alain Friboulet, Jagadeesh Bayry, Srini V. Kaveri

**Affiliations:** 1 Institut National de la Santé et de la Recherche Médicale Unité 1138, Centre de Recherche des Cordeliers, 15 rue de l’Ecole de Médicine, Paris F-75006, France; chaitrali.roy@gmail.com (C.S.); mdasmicro@gmail.com (M.D.); esvkai@gmail.com (E.S.-V.); 2 Université de Technologie de Compiègne, UMR CNRS 6022, Compiègne F-60205, France; alain.friboulet@utc.fr; 3 Centre de Recherche des Cordeliers, Immunopathologie et Immuno-Intervention Thérapeutique, Paris F-75006, France; 4 Sorbonne Universités, UPMC Univ Paris 06, UMR S 1138, Paris F-75006, France; 5 Université Paris Descartes, Sorbonne Paris Cité, UMR S 1138, Paris F-75006, France

**Keywords:** *Viscum album*, innate cells, dendritic cells, maturation, cytokines, T cell response, IFN-γ, Th17, Th1, Th2, regulatory T cell

## Abstract

Extracts of *Viscum album* (VA); a semi-parasitic plant, are frequently used in the complementary therapy of cancer and other immunological disorders. Various reports show that VA modulates immune system and exerts immune-adjuvant activities that might influence tumor regression. Currently, several therapeutic preparations of VA are available and hence an insight into the mechanisms of action of different VA preparations is necessary. In the present study, we performed a comparative study of five different preparations of VA on maturation and activation of human dendritic cells (DCs) and ensuing CD4^+^ T cell responses. Monocyte-derived human DCs were treated with VA Qu Spez, VA Qu Frf, VA M Spez, VA P and VA A. Among the five VA preparations tested VA Qu Spez, a fermented extract with a high level of lectins, significantly induced DC maturation markers CD83, CD40, HLA-DR and CD86, and secretion of pro-inflammatory cytokines such as IL-6, IL-8, IL-12 and TNF-α. Furthermore, analysis of T cell cytokines in DC-T cell co-culture revealed that VA Qu Spez significantly stimulated IFN-γ secretion without modulating regulatory T cells and other CD4^+^ T cytokines IL-4, IL-13 and IL-17A. Our study thus delineates differential effects of VA preparations on DC maturation; function and T cell responses.

## 1. Introduction

Extracts of *Viscum album* L. (VA) or European mistletoe, a semi-parasitic plant, are traditionally used for the complementary therapy of cancer and other disorders [1,2,3,4]. Several lines of evidence indicate that VA improves patient survival, reduces the damage caused by conventional cancer therapies and increases patients’ quality of life [1,5,6]. Depending on the concentration used for treatment, mistletoe extracts induce tumor cell death and exert direct necrotic effects or apoptosis [2]. VA preparation is a heterogeneous mixture of several bio-active molecules, but the major components are lectin and viscotoxin. Mistletoe lectin (ML) consists of two subunits, the A chain (29 KDa) and B chain (34 KDa). The A chain is responsible for ribosome inactivation, whereas the B chain helps in binding to terminal galactoside residues on cell membrane [7,8].

Dendritic cells (DCs) are antigen presenting (APCs) and involved in mounting and modulating the immune response. Being sentinels of the immune system, DCs bridge innate and adaptive immunity. Thus, DCs are potential targets for the therapeutic intervention in immune-mediated conditions. Immature DCs expressing low MHC II on their surface are specialized in uptake of antigens. Upon receiving activation signals, DCs undergo maturation and induce distinct CD4^+^ T cell responses. The mature DCs express high level of MHC II and co-stimulatory molecules and secrete a large array of cytokines that mediate inflammation and CD4^+^ cell polarization [9,10,11,12,13,14]. However, in the absence of danger signals, presentation of self-antigens by immature DCs promotes immune tolerance by silencing the effector and autoreactive T cells and enhancing CD4^+^CD25^+^FoxP3^+^ regulatory T cells (Tregs) or T regulatory type 1 cells [9,15,16,17,18].

As DCs have a central role in anti-tumor immune responses, efficient functioning of these cells is crucial for the success of cancer immunotherapy [19]. DCs are immature and functionally defective in cancer patients and tumor-bearing animals, possibly due to insufficient danger signals in the tumor microenvironment [20]. Further, several reports indicate that tumor cells hamper the maturation process of DCs and their capacity to prime protective T cell responses [21,22,23,24].

Our previous report demonstrates that VA Qu Spez, one of the VA preparations, induces activation of human DCs, and DC-mediated CD4^+^ T cell proliferation and tumor-specific CD8^+^ T cell responses as measured by IFN-γ and TNF-α secretion [25]. However, several therapeutic preparations of VA are currently available. Each VA preparation is heterogeneous in its chemical composition and is influenced by the host tree, harvest season and extraction method [26,27,28]. Therefore, the therapeutic outcome of a particular VA preparation might not be similar to that of other preparations [29,30]. An insight into the mechanisms of action of different VA preparations is therefore necessary to provide guidelines for the correct therapeutic use of VA preparations. 

In the present study, we performed a comparative study of five different preparations of VA (VA Qu Spez, VA Qu Frf, VA M Spez, VA P and VA A) on the maturation and activation of human DCs and ensuing CD4^+^ T cell responses. Our data show that among five preparations tested, VA Qu Spez is the most potent inducer of DC maturation and secretion of DC cytokines. Furthermore, VA Qu Spez significantly stimulated IFN-γ secretion without modulating Tregs and other CD4^+^ T cytokines IL-4, IL-13 and IL-17. Our study thus delineates differential effects of VA preparations on DC maturation, function and T cell responses.

## 2. Results

### 2.1. Effect of Different VA Preparations on the Maturation of DCs

**Figure 1:**
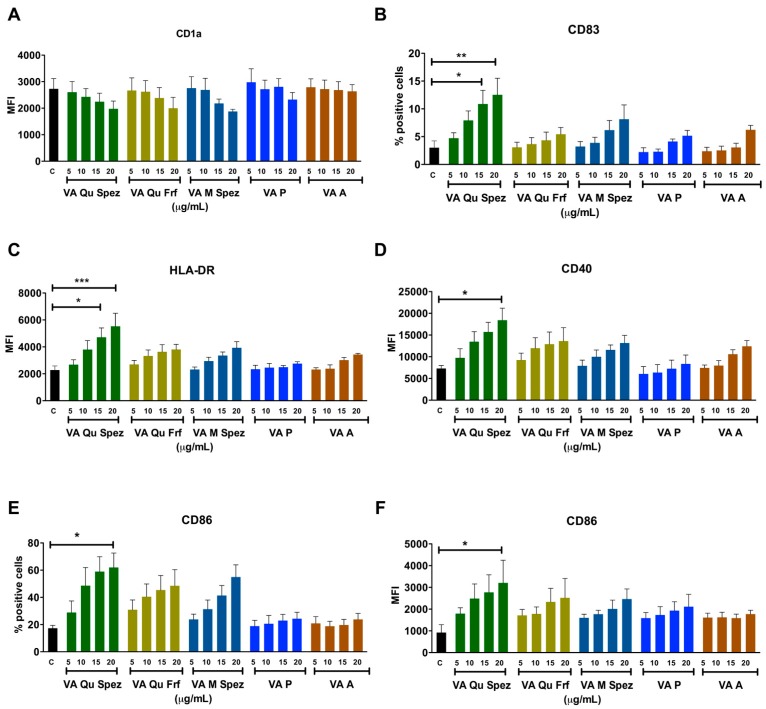
Differential effects of VA preparations on the phenotype of human DCs. Immature DCs were treated with medium alone (control, labelled as ‘C’) or with five preparations of VA (VA Qu Spez, VA Qu Frf, VA M Spez, VA P and VA A) at indicated concentrations for 48 h. Expressions (mean ± SEM, ≥4 independent donors) of (**A**) CD1a; (**B**) CD83; (**C**) HLA-DR; (**D**) CD40; (**E**,**F**) CD86 on DCs were analysed by flow cytometry. The data are presented either as % positive cells or MFI of indicated markers. X-axis denotes concentrations of VA preparations. * *p* < 0.05, ** *p* < 0.01, *** *p* < 0.001.

Immature DCs of 5 day old were either untreated or treated with five VA preparations at four different concentrations: 5, 10, 15 and 20 µg/mL/0.5 × 10^6^ cells for 48 h. DCs were analysed for the expression of various maturation-associated surface molecules (Figure 1A–F). We found that among five VA preparations, only VA Qu Spez was able to significantly enhance the intensity of expression of antigen presenting molecule HLA-DR, co-stimulatory molecules CD86 and CD40 and % of expression of terminal maturation marker CD83. The induction of DC maturation by VA Qu Spez was observed only at higher concentrations i.e., 15 and 20 µg. Further, the effect of VA Qu Spez on maturation of DCs was dose-dependent. The expressions of CD40 and HLA-DR were 100% on control DCs and were not altered by VA Qu Spez. VA Qu Spez also did not alter % expression of CD1a and intensity of expression of CD83.

We observed that HLA-DR expression on VA Qu Spez (20 µg) and LPS (positive control, 10 ng/0.5 million cells)-stimulated DCs was similar. However, induction of CD40 and CD86 by VA Qu Spez was 2-fold lesser and CD83 was 4-fold lesser than LPS. In line with our previous report on stimulation of tumor-antigen-specific cytotoxic T cell responses by VA Qu Spez-stimulated DCs [25], we found that these DCs expressed higher levels of HLA class I molecules (13.6% ± 1.1% on control DCs vs. 20.6% ± 3.2% on VA Qu Spez-stimulated DCs, *n* = 3). However, VA Qu Frf, VA M Spez, VA P and VA A did not significantly modify the expressions of any of maturation-associated molecules on DCs. These results suggest that among all preparations tested; only VA Qu Spez is able to induce maturation of DCs.

### 2.2. VA Qu Spez but Not Other VA Preparations Stimulate the Secretion of DC Cytokines

**Figure 2:**
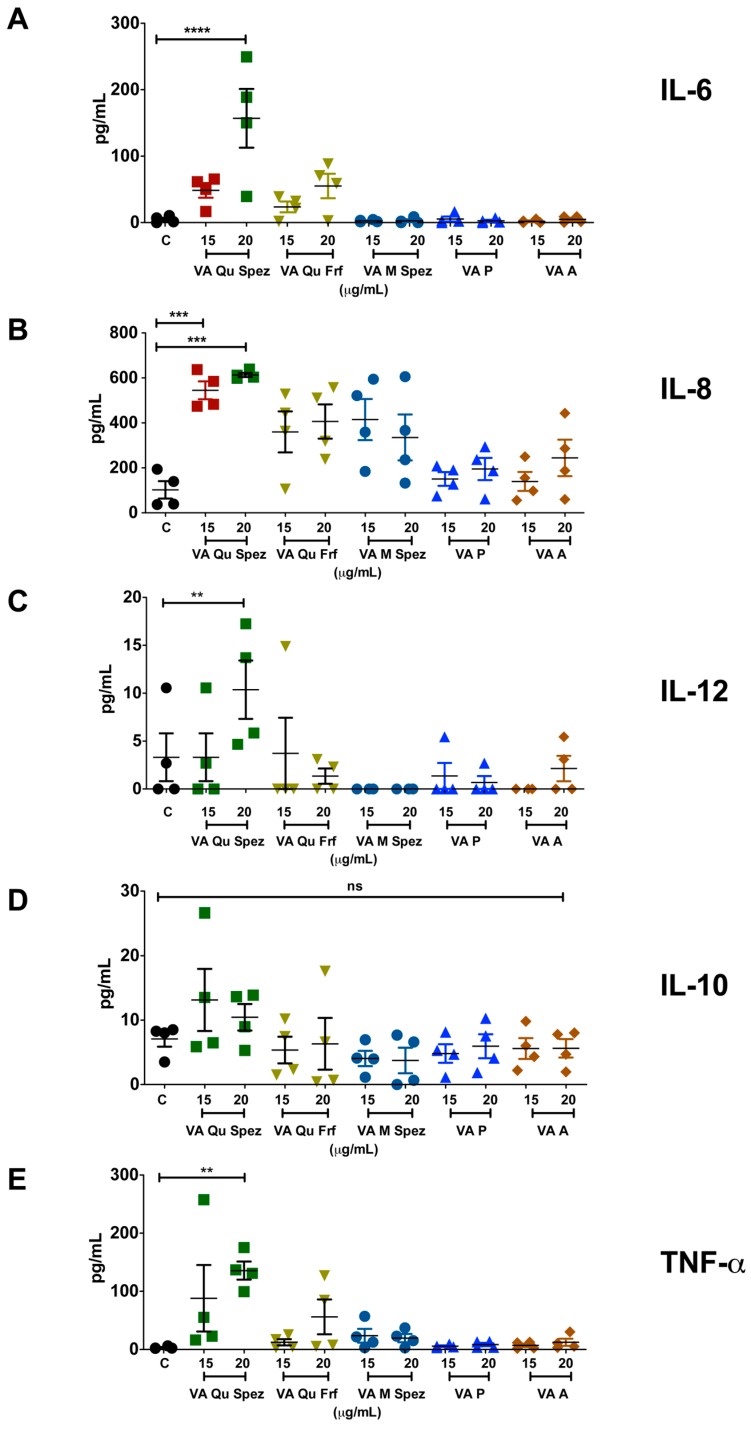
VA Qu Spez but not other VA preparations stimulate the secretion of DC cytokines. Immature DCs were untreated (control, labelled as ‘C’) or treated with five preparations of VA at various concentrations for 48 h. The amount (pg/mL, mean ± SEM, four independent donors) of (**A**) IL-6; (**B**) IL-8; (**C**) IL-12; (**D**) IL-10; and (**E**) TNF-α in cell-free supernatants was measured. ** *p* < 0.01, *** *p* < 0.001, **** *p* < 0.0001.

It is well reported that DC-derived cytokines play a critical role in regulating the immune responses and in polarizing distinct CD4^+^ T cell responses. We analysed the differential effects of various VA preparations on the secretion of DC cytokines such as IL-6, IL-8, IL-12, IL-10 and TNF-α. As VA Qu Spez significantly induced maturation of DCs, it was likely that this effect is associated with modulation of DC cytokines. In fact, compared to control DCs, VA Qu Spez-treated DCs showed significantly increased secretion of IL-6, IL-8, IL-12 and TNF-α (Figure 2A–C,E). Control DCs secreted 4.7 ± 5.1 pg/mL of IL-6 and was enhanced to 156.9 ± 105.1 pg/mL by VA Qu Spez. In case of IL-8, control DCs secreted 102.2 ± 78.5 pg/mL, whereas VA Qu Spez at the highest concentration induced 612.1 ± 20.4 pg/mL. The Th1-polarizing cytokine IL-12 was secreted at 3.3 ± 4.9 pg/mL by control DCs and was increased to 10.4 ± 6 pg/mL by VA Qu Spez-treated DCs. TNF-α secretion by untreated DCs was 3.2 ± 2.1 pg/mL, and with VA Qu Spez treatment, this cytokine was increased to 135.7 ± 37.9 pg/mL. We could observe a moderate but insignificant induction of the aforementioned DC cytokines by VA Qu Frf and VA M Spez. However, VA P and VA A did not modulate any of the DC cytokines (Figure 2A–C,E). These results show that VA Qu Spez is the most potent preparation that induces both maturation and cytokines by DCs. Of note, production of IL-10, an immunosuppressive cytokine was unaltered upon VA Qu Spez treatment (Figure 2D). Together, our data suggest that VA Qu Spez significantly induces several pro-inflammatory cytokines without modulating immune-suppressive cytokine IL-10.

### 2.3. Differential Effects of VA Preparations on the CD4^+^ T Cell Response

**Figure 3:**
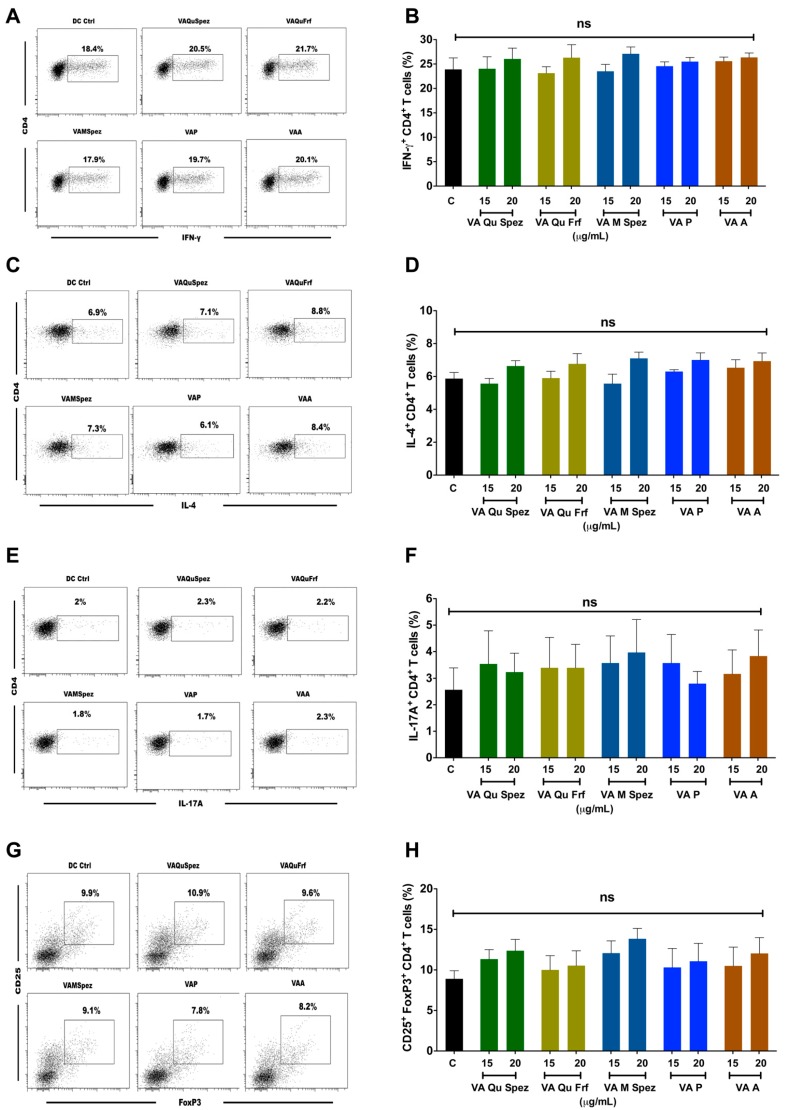
Effect of various VA preparations on the CD4^+^ T cell responses. DCs were treated with medium alone (DC Ctrl, labelled as ‘C’) or with five preparations of VA for 48 h. These DCs were co-cultured with CD4^+^ T cells at 1:10 ratio. After five days of co-culture, the cells were analysed for the various CD4^+^ T cell subsets by intra-cellular cytokines (IFN-γ, IL-4, IL-17A) or transcription factor (FoxP3) for Th1, Th2, Th17 and Tregs respectively. (**A**,**C**,**E**,**G**) representative dot plots showing the proportion of IFN-γ^+^, IL-4^+^, IL-17A^+^ CD4^+^ T cell and CD4^+^CD25^+^Foxp3^+^ T cells respectively; (**B**,**D**,**F**,**H**) Percentage (mean ± SEM, six independent donors) of IFN-γ^+^ Th1, IL-4^+^ Th2, IL-17A^+^ Th17 and CD4^+^CD25^+^Foxp3^+^ Treg cells respectively. ns, non-significant.

**Figure 4:**
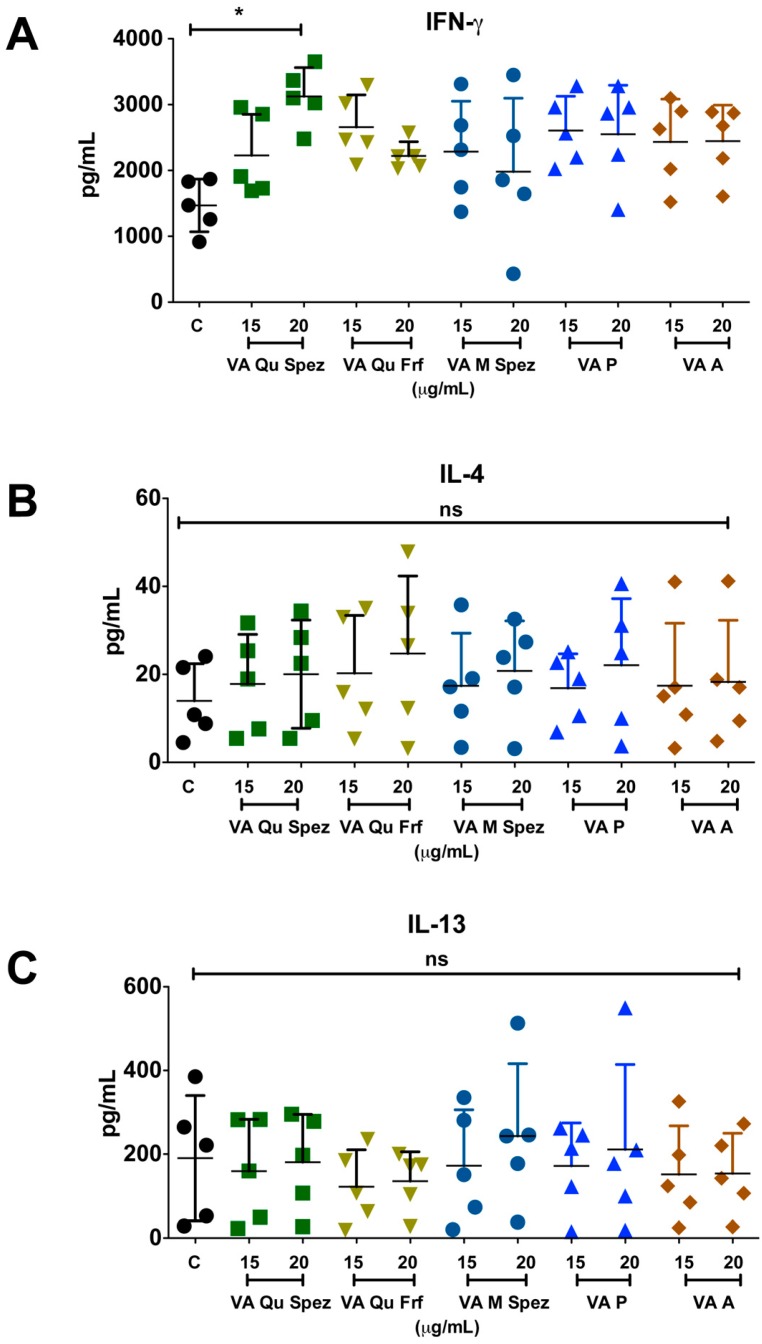

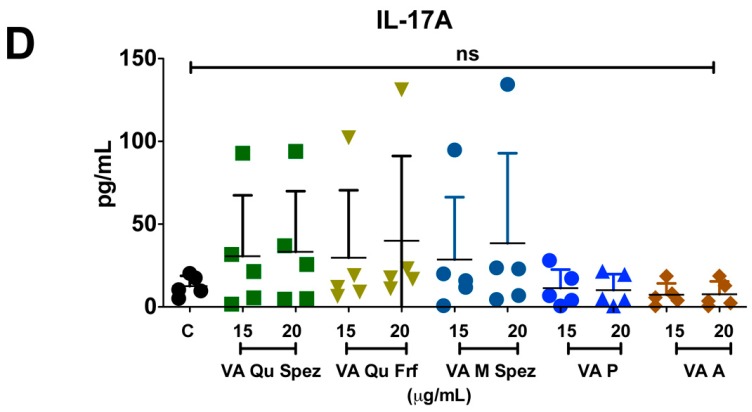
VA Qu Spez-educated DCs significantly induce the secretion of Th1 cytokine IFN-γ in DC-CD4^+^ T co-cultures. Immature DCs were treated with medium alone (control, labelled as ‘C’) or with five preparations of VA for 48 h. These DCs were co-cultured with CD4^+^ T cells for five days. Amount (pg/mL, mean ± SEM, five independent donors) of secretion of (**A**) IFN-γ; (**B**) IL-4; (**C**) IL-13; and (**D**) IL-17A in the cell-free supernatants from the above co-cultures was presented. * *p* < 0.05.

One of the key functions of APC is to promote CD4^+^ T cell responses. DCs primed with various preparations of VA were co-cultured with CD4^+^ T cells and Th1, Th2, Th17 and Treg responses were determined by flow cytometric analysis of intracellular IFN-γ (Th1), IL-4 (Th2), IL-17A (Th17), FoxP3 (Treg). Although VA Qu Spez induced maturation of DCs, this effect was not associated with the modulation of frequency of any of the T cell subsets (Figure 3A–H). However, analysis of amount of secretion of T cell cytokines in DC-CD4^+^ T cell co-culture revealed that VA Qu Spez significantly stimulated IFN-γ secretion (Figure 4A), without having any effect on the secretion of IL-4 (Figure 4B), IL-13 (Figure 4C) and IL-17A (Figure 4D). These results suggest that VA Qu Spez selectively favours Th1 responses without modulating Th2, Th17 and Treg responses. Other four preparations of VA did not alter either frequency of T cell subsets or secretion of various T cell cytokines. These results were in line with the fact that VA Qu Frf, VA M Spez, VA P and VA A did not induce maturation and activation of DCs.

## 3. Discussion

Currently available mistletoe extracts are highly heterogeneous due to differences in the host trees, nutritional source, season of harvest, and extraction methods [4,26,27,28]. Therefore, VA preparations could exert divergent biological activities. However, comparative study of immunomodulatory properties of different VA extracts on immunocompetent cells such as DCs has not been performed to date. The present data therefore provide guidelines for the therapeutic use of VA preparations. 

IFN-γ plays an important role in mediating the protective immune response against cancer, viral and intracellular bacterial infections [31]. IFN-γ enhances MHC class I expression on tumor cells and MHC class II expression on APCs like DCs, which in turn link innate and adaptive immunity [32]. IFN-γ responsiveness of tumor cell is important for the successful immune recognition. Indeed, it has been demonstrated that mice that are non-responsive to IFN-γ develop more tumors as compared to wild-type mice. Studies have shown that cross-talk between lymphocytes and IFN-γ/STAT1 signalling pathway plays an important role in maintaining the immune competiveness of the host [33]. Idiotype-specific CD4^+^ Th1 cells can achieve tumor apoptosis directly by Fas/Fas L interaction and indirectly by IFN-γ production [34]. Thus, IFN-γ pathway is considered as an extrinsic tumor-suppressor mechanism [35]. We found that VA Qu Spez significantly enhances IFN-γ production without modulating Treg subsets and production of other T cell cytokines IL-4, IL-13 and IL-17A. This selective enhancement of Th1 cytokine strongly supports the use of VA as an immune modulator. 

The success of DC-based cancer immunotherapies is dependent on the maturation status of DCs, their migration capacity and ability to mount protective T cell responses [36]. DC immunotherapy for cancer in humans though shown promises, it has not met with great success as compared to therapeutic molecules that target immune checkpoints. The reasons are multiple including poor survival of transferred DCs, limited number of DCs reaching the secondary lymphoid organs, heterogeneity in the DC subtypes and immune suppressive environment created by the tumor. Previous reports have shown that PGE_2_ produced by DCs mediate Treg expansion [37,38,39], which might help in tumor evasion. Vaccination of cancer patients with ‘PGE_2_-educated DCs’ also induced Treg expansion in the patients [40]. We observed that VA Qu Spez did not modulate Treg responses suggesting that VA Qu Spez selectively induces IFN-γ responses. Although not examined in DCs, we have recently shown that VA Qu Spez inhibits COX2-mediated PGE_2_ in epithelial cell line [41,42]. Therefore, it is likely that VA Qu Spez-mediated suppression of COX-2 in DCs might be responsible for nonmodulation of Tregs in the present study. As these data are from the in vitro experiments, further work is necessary to validate these results from the patients treated with VA. Of note, through enhancement of Fas/FasL expression and caspase activation, IFN-γ has been shown to enhance apoptotic response to ML II in human myeloid U937 cells [43]. 

MLs are the active components of mistletoe extracts and have several functions. The cytotoxicity of mistletoe is attributed majorly to its lectin contents [44,45] and lectin internalization is required for ML-I-mediated apoptosis [46]. MLs are responsible for stimulating cells of the innate and adaptive immune system such as DCs, macrophages, natural killer cells, and B and T lymphocytes. This function of MLs might represents one of the mechanisms responsible for the anti-tumoral and immunomodulatory effects of mistletoe extracts. It is known that ML-I B chain causes Ca^2+^ influx in Jurkat cells and is mediated by its interaction with surface glycoprotein receptors [47]. Chemical labelling of the lectin revealed that it binds to surface of peripheral and intra-tumoral monocytes [48].

A recent study shows that 3D structure of ML-A chain shares structural homology with shiga toxin from *Shigella dysenteriae* and provides an explanation for the strong immune stimulatory capacity of ML [49]. It is also demonstrated that Korean mistletoe lectin (KML) induces activation of innate cells by TLR4-mediated signalling [50]. The nature of the receptor(s) on DCs that recognizes ML and mediates activation is not known. Since Korean ML and European ML share 84% sequence identity [51], it is presumable that European ML might signal DCs via TLR [49]. However, we found that not all VA preparations are stimulatory on DCs. VA Qu Frf, an unfermented preparation containing the highest concentration of lectin and viscotoxin was unable to activate DCs. Other VA preparations, which are fermented and contain low lectin, were also unable to stimulate DCs, whereas VA Qu Spez, a fermented preparation that contains the second highest concentration of lectin (785 ± 10% ng/mL) efficiently activated DCs and promoted Th1 response. These results suggest that mere lectin content in a VA preparation does not necessarily determine its immunostimulatory capacity. The methodology of preparation, i.e., fermented vs unfermented, might be crucial for conferring the stimulatory properties to VA. Alternatively, the fermentation process might modify the structure of the lectins of the VA preparation.

To conclude, our study delineates the differential effects of various VA preparations on DC maturation, function and T cell responses. These results reveal that VA Qu Spez is the most potent preparation in activating DCs and promoting Th1 response. The current evidence to support mistletoe therapy in oncology is weak [52]. Thus, this study along with other reports on mistletoes [53,54,55,56,57,58,59,60] provides a rational for examining the use VA as an immune modulator. Such mechanistic studies are also important to undertake randomised clinical trials to improve level of evidence for the use of VA in complementary therapy of cancer.

## 4. Materials and Methods

### 4.1. VA Preparations

Five clinical grade preparations of VA (VA Qu Spez, VA Qu Frf, VA M Spez, VA P and VA A) obtained from Hiscia Institute, Verein für Krebsforschung (Arlesheim, Switzerland) were used. These preparations were free from endotoxins and were formulated in 0.9% sodium chloride isotonic solution as 5 mg/mL vials. The chemical compositions of the VA preparations are provided in Table 1.

### 4.2. Human DCs

Human monocyte-derived DCs were used as a source of DCs. Peripheral blood mononuclear cells (PBMC) were isolated from buffy coats of healthy donors. The buffy coats were purchased from Centre Necker-Cabanel (EFS, Paris, France). Ethics committee approval for the use of such material (Institut National de la Santé et de la Recherche-EFS Ethical Committee Convention N°12/EFS/079) was obtained and experiments were performed in accordance with the approved guidelines of INSERM. Circulating monocytes were isolated using CD14 microbeads (Miltenyi Biotec, Paris, France) and were cultured for 5 days in RPMI 1640 containing 10% fetal calf serum, rhIL-4 (500 IU/10^6^ cells) and rhGM-CSF (1000 IU/10^6^ cells) to obtain immature DCs [61].

### 4.3. Viscum Album Treatment of DCs

Immature DCs were washed and cultured in rhIL-4 and rhGM-CSF and treated with VA Qu Spez, VA Qu Frf, VA M Spez, VA P and VA A at four different concentrations: 5, 10, 15 and 20 µg/mL/0.5 million cells for 48 h. Cell culture supernatants were collected for analysing the cytokines and DCs were analysed for the phenotype by flow cytometry.

### 4.4. DC: CD4^+^ T Cell Co-Cultures

CD4^+^ T cells were isolated from the PBMC using CD4 microbeads (Miltenyi Biotec). VA-treated DCs were washed extensively and seeded with 1 × 10^5^ responder allogeneic CD4^+^ T cells at DC: T cell ratio of 1:10. On 5th day, CD4^+^ T cell responses were analysed by intra-cellular staining for specific T cell cytokines (IFN-γ, IL-17A and IL-4) and transcription factor (FoxP3). The cell-free culture supernatants were analysed for the cytokines secreted.

### 4.5. Flow Cytometry

For surface staining, following Fc receptor blockade, antibodies against surface molecules were added at pre-determined concentration and incubated at 4 °C for 30 min. FITC-conjugated monoclonal antibodies (MAbs) to CD1a, CD86, HLA-DR, and CD25; PE-conjugated MAbs to CD83 (all from BD Biosciences, Le Pont de Claix, France), CD40 (Beckman Coulter, Villepinte, France) and Alexa Fluor^®^ 700-conjugated MAbs to CD4 (eBioscience, Paris, France) were used for the analysis of surface phenotype.

For intra-cellular staining, cells were stimulated with phorbolmyristate acetate (50 ng/mL; Sigma-Aldrich, St. Quentin Fallavier, France) and ionomycin (500 ng/mL; Sigma-Aldrich) at 37 °C for 5–6 h in the presence of golgi-stop (BD Biosciences) during the last 2 h. Cells were fixed and permeabilised using Foxp3 Fixation/Permeabilization kit (eBioscience) and incubated at 4 °C with FITC-conjugated MAbs to IFN-γ (eBioscience), PE-conjugated MAbs to IL-17A and IL-4 (eBioscience), and APC-conjugated MAbs to FoxP3 (eBioscience). Live-dead cells were differentiated by PO-Fixable Viable dye (eBioscience).

Cells were acquired on LSR II and processed with FACS DIVA software (BD Biosciences) and analysed by Flowjo. The data were presented as % positive cells for indicated markers or mean fluorescence intensities (MFI) of their expression.

### 4.6. Cytokine Assay

IL-6, IL-8, IL-10, IL-12, TNF-α, IL-4, IL-13, IFN-γ and IL-17A in the cell-free culture supernatants were quantified by Ready-SET-Go enzyme-linked immunosorbent assay (ELISA) kits (eBioscience).

### 4.7. Statistical Analysis

The significant differences between samples were determined by One-way ANOVA Tukey’s multiple comparison test using Prism 5 software (GraphPad Software Inc., La Jolla, CA, USA). Values of *p* < 0.05 were considered statistically correlated (* *p* < 0.05, ** *p* < 0.01, *** *p* < 0.001, **** *p* < 0.0001). 

## 5. Conclusions

Our study demonstrates the differential effects of various VA preparations on human DC activation and ensuing CD4^+^ T cell responses. Our data reveal that VA Qu Spez is the most potent VA preparation in activating DCs and promoting Th1 response. 

## Figures and Tables

**Table 1:** Composition of VA preparations.

Preparation Concentration	Host Trees	Lectin Content (ng/mL)	Viscotoxin Content (µg/mL)	Method of Preparation
VA Qu Spez 10 mg	Quercus (Oak)	785 ± 10%	5 ± 5%	Fermented
VA Qu Frf 10 mg	Quercus (Oak)	2391 ± 10%	19 ± 5%	Unfermented
VA M Spez 10 mg	Malus (Apple)	548 ± 10%	4 ± 5%	Fermented
VA P 10 mg	Pinus (Pine)	28 ± 10%	6 ± 5%	Fermented
VA A 10 mg	Abies (Fir)	23 ± 10%	19 ± 5%	Fermented
