# Correction: Saha, C., et al. Differential Effects of *Viscum album* Preparations on the Maturation and Activation of Human Dendritic Cells and CD4^+^ T Cell Responses. *Molecules* 2016, *21*, 912

**DOI:** 10.3390/molecules24203762

**Published:** 2019-10-18

**Authors:** Chaitrali Saha, Mrinmoy Das, Emmanuel Stephen-Victor, Alain Friboulet, Jagadeesh Bayry, Srini V. Kaveri

**Affiliations:** 1 Institut National de la Santé et de la Recherche Médicale, F-75006 Paris, France; chaitrali.roy@gmail.com (C.S.); mdasmicro@gmail.com (M.D.); esvkai@gmail.com (E.S.-V.); 2 Université de Technologie de Compiègne, UMR CNRS 6022, F-60205 Compiègne, France; alain.friboulet@utc.fr

The authors wish to make the following corrections to this paper [1]:

Two dot-plots in the Panel E of Figure 3 (labeled under VAP and VAA) have been inadvertently duplicated during the final preparation of figures. We would like to change the Panel E of Figure 3 in paper [1] to the correct version, as follows:

We apologize for this unintentional mistake, which, however, does not affect the results of this manuscript and the conclusions drawn from them.

## Figures and Tables

**Figure 3:**
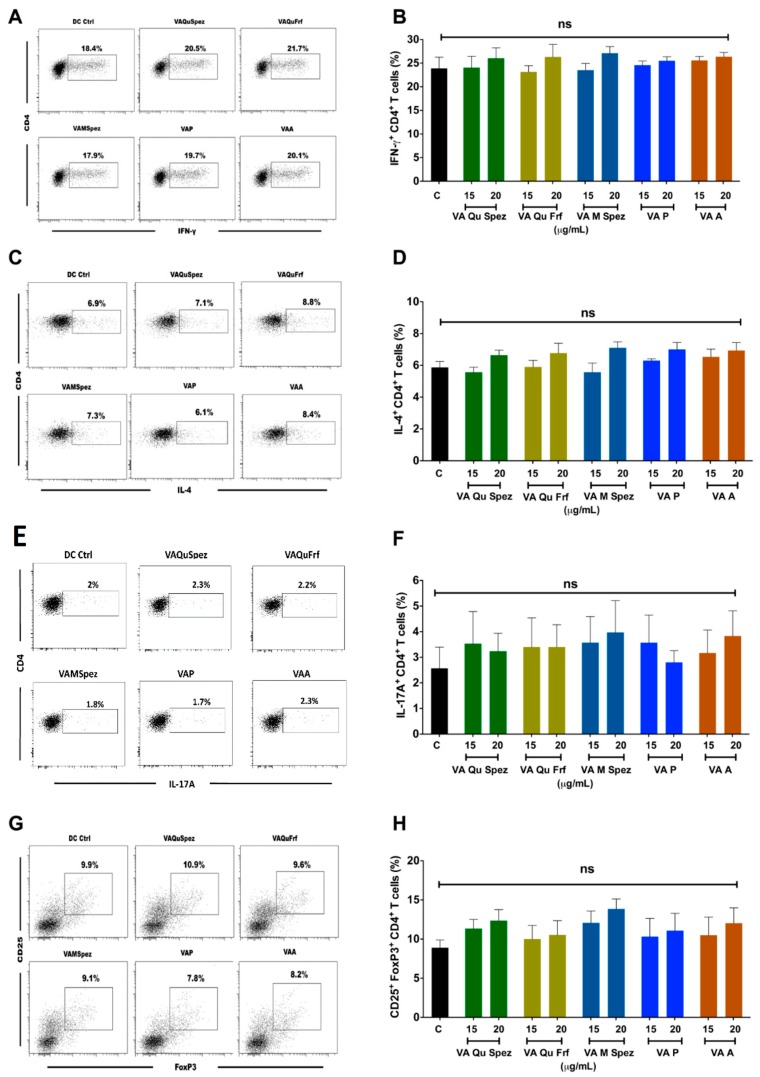
Effect of various VA preparations on the CD4^+^ T cell responses. DCs were treated with medium alone (DC Ctrl, labelled as ‘C’) or with five preparations of VA for 48 h. These DCs were co-cultured with CD4^+^ T cells at 1:10 ratio. After five days of co-culture, the cells were analysed for the various CD4^+^ T cell subsets by intra-cellular cytokines (IFN-γ, IL-4, IL-17A) or transcription factor (FoxP3) for Th1, Th2, Th17 and Tregs respectively. (**A**,**C**,**E**,**G**) representative dot plots showing the proportion of IFN-γ^+^, IL-4^+^, IL-17A^+^ CD4^+^ T cell and CD4^+^CD25^+^Foxp3^+^ T cells respectively; (**B**,**D**,**F**,**H**) Percentage (mean ± SEM, six independent donors) of IFN-γ^+^ Th1, IL-4^+^ Th2, IL-17A^+^ Th17 and CD4^+^CD25^+^Foxp3^+^ Treg cells respectively. ns, non-significant.
